# Unique Case of Rare Non-Neural Granular Cell Tumor of the Rectus Abdominis Muscle

**DOI:** 10.3390/medicina60040576

**Published:** 2024-03-31

**Authors:** Petar Ivanov Kiskinov, Anastas Metaxov Palavurov, Angelina Yanakieva Mollova-Kyosebekirova, Kiril Todorov Atliev, Elean Ivanov Zanzov, Vania Nikolaeva Anastasova

**Affiliations:** 1 Department of Propaedeutics of Surgical Diseases, Section of Plastic, Reconstructive and Aesthetic Surgery and Thermal Trauma, Medical University Plovdiv, “Saint George” University Hospital, 4002 Plovdiv, Bulgaria; zanzov@gmail.com (E.I.Z.); vania_anastasova@yahoo.com (V.N.A.); 2 Department of Urology and General Medicine, Medical University Plovdiv, “Saint George” University Hospital, 4002 Plovdiv, Bulgaria; anastas1112@abv.bg (A.M.P.); atlievamaria@gmail.com (K.T.A.); 3 Department of General and Clinical Pathology, Medical University of Plovdiv, “Saint George” University Hospital, 4002 Plovdiv, Bulgaria; angelinamollovakyosebekirova@gmail.com

**Keywords:** Abrikossoff’s tumor, granular cell tumor, rectus abdominis muscle, pregnancy

## Abstract

*Background and Objectives*: Our report contributes a unique case of a non-neural GCT occurring in an unusual location, with its development during pregnancy adding to its rarity. *Materials and Methods*: Granular cell tumors (GCTs), also known as Abrikossoff’s tumors, are rare neoplasms of Schwann cell origin with predominantly benign behavior. We present a case of a 29-year-old female with a non-neural variant of a GCT discovered incidentally during a cesarean section, situated on the posterior surface of the rectus abdominis muscle. *Results*: Histologically, the tumor exhibited features consistent with a benign non-neural GCT, confirmed through an immunohistochemical analysis. Despite the atypical presentation and challenging surgical removal due to prior scarring, the patient experienced no postoperative complications and showed no signs of recurrence during follow-up. *Conclusions*: This case highlights the importance of considering GCTs in differential diagnoses, particularly in unusual anatomical locations, and underscores the favorable prognosis associated with timely surgical intervention.

## 1. Introduction

The granular cell tumor (GCT), also called Abrikossoff’s tumor, is a rare finding with a good prognosis and Schwann cell origin. It can occur at any age but occurrence in children under 5 years old is uncommon. In the pathology literature, there is evidence about higher incidence among females. It is more prevalent in individuals with darker skin pigmentation compared to those with lighter skin tones, with a ratio of 3:1 [1]. Any part of the body can be affected, with a little predominance on the head and neck. Treatment is surgical with a wide excision [2].

The non-neural variant of granular cell tumors exhibits a similar morphology to its neural counterpart but lacks features of Schwann cell differentiation. Approximately 75 cases of this variant have been documented in the literature over the past 30 years. While the tumor primarily affects the skin of the limbs and back, occurrences have also been reported on the face and scalp. Interestingly, the most common non-cutaneous location is the oral cavity [3,4,5,6]. Due to the wide range of possible differential diagnoses, the final diagnosis of non-neural granular cell tumors typically requires a thorough histological and immunohistochemical examination [6].

## 2. Materials and Methods

Herein, we present a detailed clinical case of a nulliparous 29-year-old female patient who presented with a newly discovered tumor formation during a caesarean section, following an uneventful first pregnancy. In our case, the patient had a normal BMI and no family history of congenital disorders or metabolic diseases among her relatives. Prior to childbirth, she did not report any complaints or symptoms. There were no palpable masses detected in her lower abdominal wall before the cesarean section. Our thorough preoperative assessment encompassed a comprehensive review of the patient’s medical history, including any previous surgeries, as well as an evaluation of her current health status and any potential risk factors associated with the surgical procedure. Additionally, extensive imaging studies were conducted to accurately characterize the nature and extent of the tumor formation and to aid in surgical planning at the second rehospitalization.

## 3. Results

The tumor is situated on the posterior surface of the rectus abdominis muscle, approximately 5 cm lateral to the midline, with extension to the bladder wall. Macroscopically, the tumor appears solid with a granular surface, exhibiting a pale pink coloration and measuring 4 × 2.5 × 1 cm in size. A biopsy was taken during the operative delivery, and the radical removal of the lesion was not attempted, because it was an incidentaloma. The histological result was a granular cell myoblastic tumor, Abrikossoff’s tumor, without malignant transformation. Follow-up imaging right after the cesarean section revealed no enlargement of the regional lymph nodes.

The baby was delivered via elective cesarean section at 39 weeks of gestation. There were no signs of distress, and the baby had an optimal Apgar score of 9/10, demonstrating good adaptation after birth.

**Figure 1:**
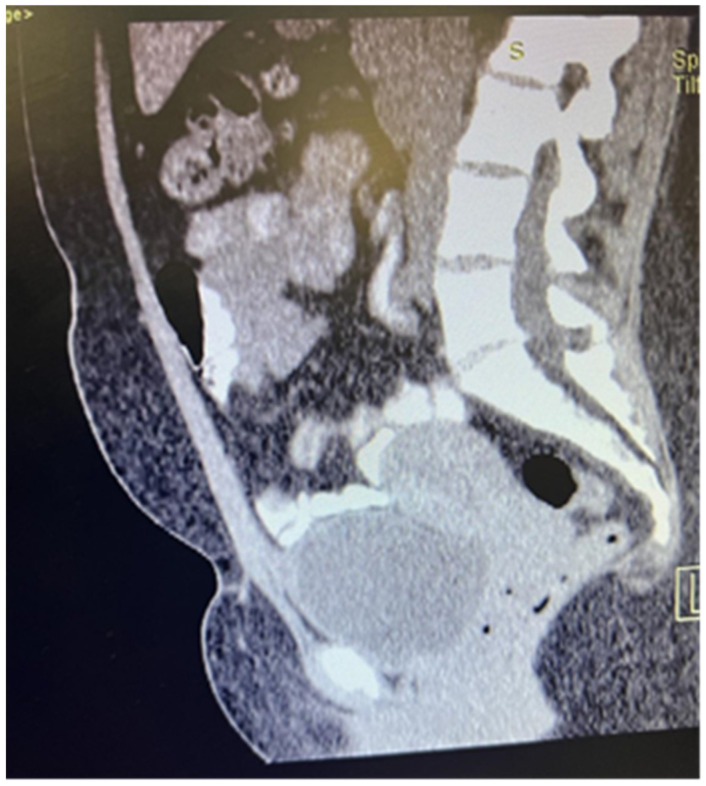
Computed tomography in sagittal section.

**Figure 2:**
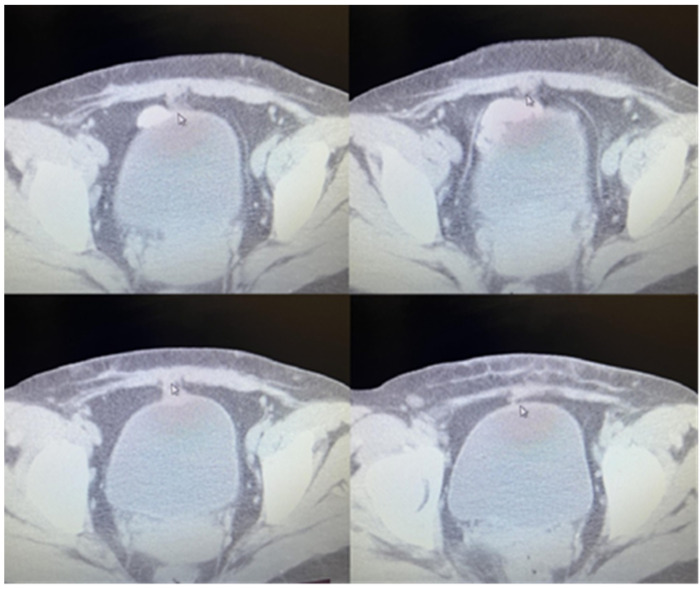
Computed tomography in transverse sections.

Due to the non-life-threatening nature of the initial biopsy result, as well as the newborn, the patient decided to postpone surgical removal. Four months following the initial biopsy, she presented herself once again, now fully prepared. A CT scan of the abdomen revealed thickening of the anterior abdominal wall, particularly in the area adjacent to the bladder, measuring up to 10 mm ventrally. A lesion, approximately 16 mm by 23 mm in size, was identified in the same area, involving the bladder wall. The lesion exhibited an irregular shape and lacked sharp outlines. The described findings are illustrated in Figure 1 (sagittal section) and Figure 2 (transverse sections).

Before proceeding with the operation, a comprehensive assessment of the patient was conducted, to meticulously identify and scrutinize any potential contraindications for surgical intervention. With the patient under general anesthesia and endotracheal intubation to ensure airway management, we commenced the procedure with a lower-middle laparotomy approach. Intraoperatively, as the incision was made through the layers of the skin, subcutaneous tissues, and the anterior aspect of the rectus abdominis fascia, meticulous examination revealed no discernible signs of pathological processes involving the surrounding tissues. However, as we delved deeper into the exploration of the dorsal aspect of the rectus abdominis and the posterior aspect of the rectal fascia, a patchy, soft, gray-whitish lesion was identified. Its removal posed considerable challenges due to the presence of abundant cicatricial tissue resulting from the patient’s previous cesarean section. Despite the lesion exhibiting a propensity to tear apart easily, with careful and gentle maneuvers, we successfully excised it, ensuring margins were free from any residual tissue. The excision encompassed the posterior part of the rectus sheath en bloc with the endopelvic fascia and a small amount of adjacent adipose tissue bordering the bladder wall. Macroscopically clear margins were achieved. Although the rectus abdominis muscle was involved, its total thickness was not significantly compromised, thereby obviating the necessity for the preoperatively planned reconstructive procedures. Subsequently, we meticulously closed all layers sequentially, ensuring there was no undue tension, and without necessitating the use of artificial materials for reinforcing the abdominal wall.

At our hospital, all specimens are routinely fixed in 10% buffered formalin and embedded in paraffin for histological evaluation. Standard 4 μm-thick sections were cut from paraffin blocs. Sections were stained with haematoxylin–eosin (HE).

**Figure 3:**
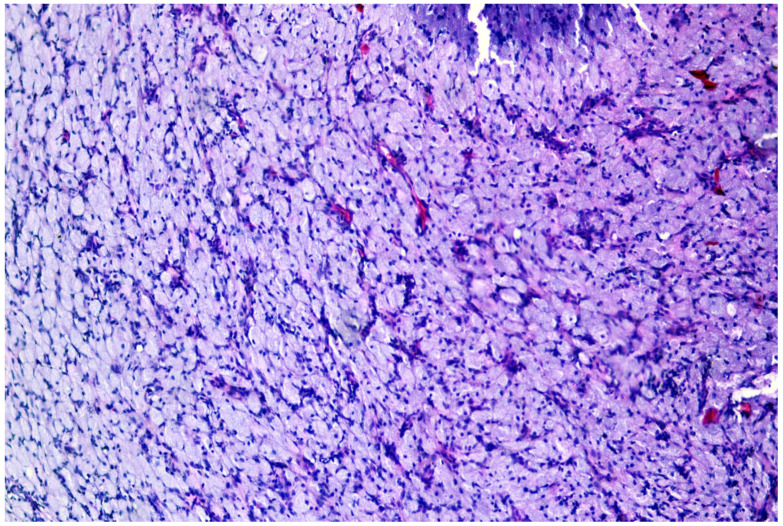
Nonneural granular cell tumor: granular cells arrayed in sheets.

**Figure 4:**
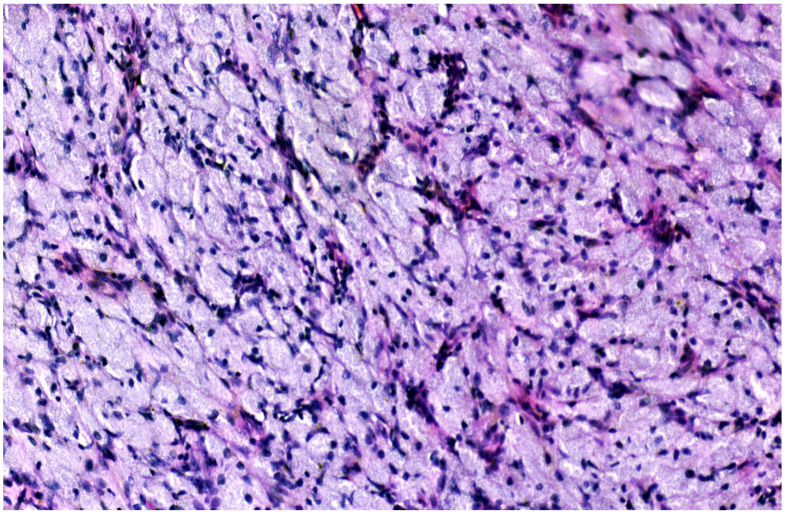
Polygonal cells with small nuclei, inconspicuous nucleoli and abundant granular eosinophilic cytoplasm.

**Figure 5:**
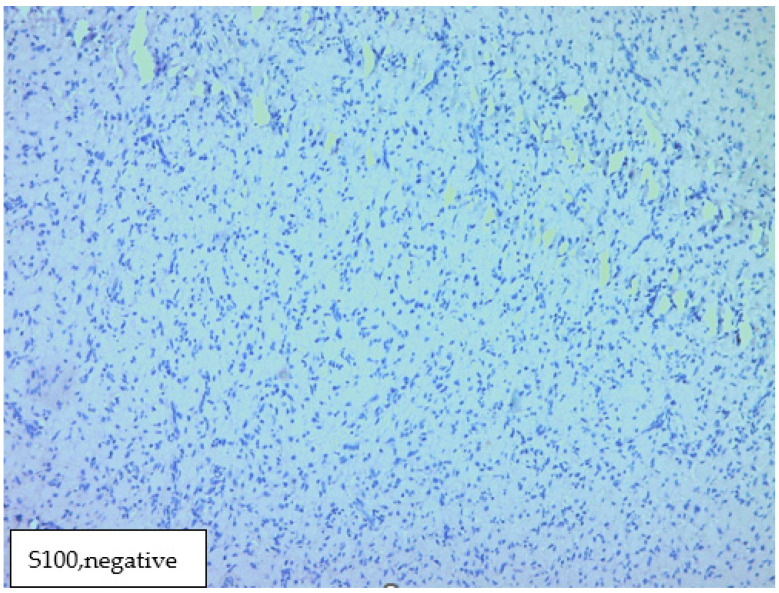
Immunohistochemical analysis showed that the cells were negative for S100.

**Figure 6:**
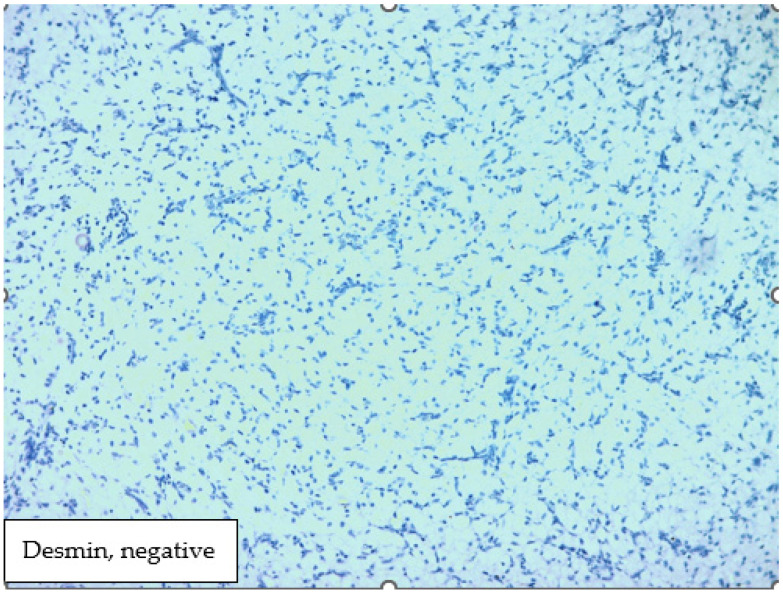
Immunohistochemical analysis showed that the cells were negative for Desmin.

**Figure 7:**
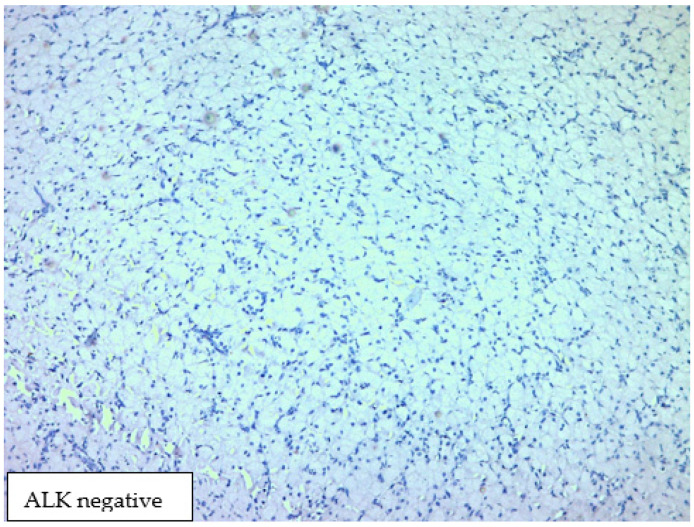
Immunohistochemical analysis showed that the cells were negative for ALK.

The histological result revealed fragments of a lesion composed of oval cells exhibiting eosinophilic granulated cytoplasm and centrally located nuclei, many of which contained eosinophilic nucleoli (Figure 3 and Figure 4). An immunohistochemical analysis showed that the cells were negative for S100, Desmin, and ALK (Figure 5, Figure 6 and Figure 7). The combination of morphological features and immunohistochemical results supports the diagnosis of a benign non-neural granular cell tumor. The patient was discharged on the fifth postoperative day without complications.

## 4. Discussion

The granular cell tumor was initially described by the Russian anatomist and pathologist, Abrikossoff, in 1926. He made significant contributions to various fields, including the study of pathomorphology in tuberculosis and tumors. The majority of his published work focuses on specialized pathology. He described for the first time a neuroectodermal tumor named by him: “myoblastoma”, which was later named Abrikossoff’s tumor in his honor [7].

Macroscopically, Abrikossoff’s tumors appear as single or multiple painless nodules with a firm consistency. Histologically, GCTs exhibit large granular cells arranged in clusters. Bands of connective tissue are observed, separated from surrounding areas by dense fibrous proliferative tissue with a pseudoepitheliomatous surface, which may be mistaken for a squamous cell carcinoma [8]. Distinguishing between benign and malignant Abrikossoff’s tumors is challenging due to their histological similarity and the absence of reliable prognostic criteria for tumor development. The ulceration of the tumor is a characteristic feature of malignant transformation. In rare cases, Abrikossoff’s tumors may be mistaken for neurofibromas or schwannomas.

The cells of granular cell tumors (GCTs) contain numerous eosinophilic cytoplasmic granules that stain positively with hematoxylin and eosin [9]. Granular cell tumors (GCTs) are classified into two types: neural GCTs (NGCTs), also known as conventional GCTs, which stain positive for S-100, and non-neural GCTs (NNGCTs), which do not [10]. Despite being relatively rare compared to other neoplastic processes, granular cell tumors (GCTs) exhibit various localizations, including atypical forms. For example, two cases of pulmonary NNGCTs have been reported in the literature [3,10]. A single case of a NGCT occurring in the uterine body of a fifty-five-year-old premenopausal patient has been documented in the literature [6]. The first researchers who described GCTs without a Schwann cell origin were P. LeBoit et al. during 1991. In their work, they described seven uncommon cutaneous granular-cell lesions, four of which were of previously undescribed origin. The suggested term for these lesions was the “primitive polypoid granular-cell tumor”. One of those tumors occurred in a child, and three were in adults. The authors concluded that further instances and extended monitoring might be necessary to eliminate the potential for the primitive polypoid granular cell tumor to be classified as a low-grade malignancy [11].

Most granular cell tumors exhibit a dense, solid morphology, characterized by slow growth and a low tendency to ulcerate. Approximately 5% of described cases exhibit a multicentric location, while less than 1% demonstrate malignancy [12]. Despite the predominance of benign variants, granular cell tumors often recur locally, posing significant challenges in patient management.

Macroscopically, the tumor we describe resembles previously published variants. However, its atypical localization distinguishes it from typical cases. Previous cases involving the anterior abdominal wall have been reported. Lee J. McGhan et al. documented a granular cell tumor originating from the subcutaneous fat tissue in this region. In the cited case, the lesion was closely associated with the fascia of the rectus abdominis muscle but did not penetrate it [13]. In our case, the tumor involved the muscle fibers, necessitating the removal of part of the muscle tissue along with a portion of the posterior fascia. Bandyopadhyay described Abrikossoff’s tumor on a BCG vaccine scar [14]. Other authors have also described GCTs of the female reproductive system [15]. To this date, there are under 20 cases of GCTs involving the abdominal wall. In their literature review, Mangan et al. state that the average age of patients with abdominal wall GCTs is 51.4 years. Furthermore, it occurs far more commonly in females than in males, with 86.7% (13 out of 15) of cases reported in females [16]. Surgical intervention, including a wide excision and histological verification of the tumor and resection margins, is essential for all patients. Regular follow-up is imperative to monitor for recurrence.

In our case, routine examinations during the pregnancy were conducted according to the protocol in the Republic of Bulgaria, and the results were satisfactory. There was no evidence of tumor growth or formation during this period. Furthermore, there is no indication of recurrence in our case. We monitored both the mother and the baby for one year following the procedure, and no signs of tumor recurrence or any abnormalities in the baby were observed.

Malignant degeneration with metastases is described in 1–3%. Gokaslan described such a malignant form with ulceration of the thigh [17]. Metastases are of increasing atypicality of the cells [18]. A malignant granular cell tumor of the abdominal wall is described by Chelly et al. [19]. Ravich et al. report a rare case of a malignant granular cell tumor involving the urinary bladder without metastases at the time of diagnosis and surgical removal [20]. Gamboa, reviewing the clinical and histological features of a case series of ten malignant GCTs, concluded that the malignant forms could be classified into two categories: clinically and histologically malignant, and clinically malignant but histologically benign. Histological evidence of visible tumor infiltration into the tissue surrounding the tumor is a regular finding in malignant lesions [21].

The largest study discussing the clinicopathological features of GCTs known in the literature was conducted by Lack et al., involving 118 granular cell tumors (GCTs) in 110 patients. Due to their varied locations, complete excisions were performed in 24 out of 56 patients who were adequately followed up. Local recurrence was observed in only five of these 24 patients. Throughout the study, the authors did not encounter any cases exhibiting malignant behavior [22].

It may be difficult to distinguish malignant from benign forms with light microscopy.

Malignant lesions are extremely rare with rapid deeply infiltrative growth and tend to recur quickly after the excision. The only reliable histopathological sign that can predict a malignant potential is the presence of metastases, increased mitotic rate, or necrosis [1,23].

Fanburg and Smith proposed a classification to evaluate the malignancy of these tumors including the following: the presence of necrosis, spindling, vesicular nuclei with large nucleoli, >2 mitoses/10 high-power fields at ×200 magnification, high nuclear-to-cytoplasmic ratio, and pleomorphism. Tumors that lack any of these criteria were considered benign, those that met one or two criteria were considered atypical, and those with three or more were classified as malignant [24]. From a histopathological point of view, the main differential diagnoses are melanocytic neoplasms, leiomyosarcoma, atypical fibroxanthomas, dermatofibroma, xanthoma, reticulohistiocytoma, adult type rhabdomyoma, and alveolar soft part sarcoma. All of these can be excluded by their histological features and immunohistochemical staining.

Melanocytic neoplasms usually have an intraepidermal component and contain melanin pigment. Most appear papule or macule-like, tan-brown, and uniformly pigmented. They are positive for HMB45 and MelanA.

Leiomyosarcomas are malignant mesenchymal tumors, showing spindle cell proliferations forming rough bundles and fascicles with nuclear pleomorphism, necrosis, and increased mitotic activity.

The atypical fibroxanthoma is a rare low grade malignant cutaneous tumor, composed of irregularly arranged spindled to epithelioid cells with no necrosis, and lymphovascular or perineural invasion. Histologically, the cells can be highly bizarre and atypical but lack connection to the overlying epidermis.

Dermatofibromas are benign intradermal lesions with fibroblastic and histiocytic differentiation. Often, they have a component of polymorphous fibroblastic or dendritic cells and collagen trapping at the periphery.

Reticulohistiocytoma, formerly called lipoid dermatoarthritis, is a rare disorder, with widespread cutaneous papules and nodules. Microscopically, the lesion is composed of histiocytic cells with abundant eosinophilic or basophilic ground glass cytoplasm.

Xanthomas are benign nodular lesions, usually associated with hyperlipidemia. Histologically, xanthomas are composed of cells with abundant pale foamy cytoplasm without atypia.

Adult type rhabdomyomas are benign tumors of the mature skeletal muscle, composed of sheets of large, well differentiated skeletal muscle cells with abundant eosinophilic fibrillar or granular cytoplasm. The tumor is positive for desmin, actin, and myogenin.

Alveolar soft part sarcoma is a rare malignant mesenchymal neoplasm of uncertain histogenesis, predominantly affecting the deep soft tissues of the extremities in young adults. It is composed of large, polygonal cells with abundant eosinophilic cytoplasm and a nested or pseudo alveolar growth pattern. Uncommon features for the tumor are nuclear pleomorphism, giant cells, mitotic figures, and necrosis [25,26].

## 5. Conclusions

This case underscores the critical importance of considering GCTs in differential diagnoses, particularly when encountered in unusual anatomical locations. Moreover, it highlights the favorable prognosis associated with timely surgical intervention. Our published case stands out significantly due to its rarity on several fronts. The atypical localization and the occurrence of the GCT during pregnancy add layers of uniqueness to this case. We observed the reduction in tumor size from the time of operative delivery to complete surgical removal, coupled with the absence of recurrence evident in follow-up imaging studies; these observations further accentuate the novelty and favorable outcomes associated with this case.

In the realm of medical literature, where cases of non-neural GCTs are sparsely documented, our report represents a valuable addition, shedding light on a previously underexplored aspect of this condition. Its exceptional circumstances and successful management serve as a testament to the importance of meticulous clinical observation, thorough diagnostic evaluation, and timely intervention in ensuring optimal patient outcomes.

## Data Availability

No new data were created or analyzed in this study. Data sharing is not applicable to this article.

## References

[1] Sposto M.R., Navarro C.M., de Andrade C.R. (2006). Granular cell tumour (Abrikossoff’s tumour): Case series. Oral Oncol. Extra.

[2] Pushpa G., Karve P.P., Subashini K., Narasimhan M.N., Ahmad P.B. (2013). Abrikossoff’s tumor: An unusual presentation. Indian J. Dermatol..

[3] Kim B.-G., Song J.Y., Zo S., Im Y., Choi S., Han J., Jeong B.-H., Kim H. (2020). S-100-Negative malignant pulmonary granular cell tumor: A case report. Respir. Med. Case Rep..

[4] Kabir B., Ramien M., Al Shammary M., de Nanassy J., El Demellawy D. (2018). Dermal non-neural granular cell tumor in a 3-year-old child. Pediatr. Dermatol..

[5] Di J., Qasem S.A. (2022). Primitive non-neural granular cell tumor: Literature review. Hum. Pathol. Rep..

[6] Deguchi Y., Iwahashi N., Horiuchi Y., Ikejima M., Tanaka T., Ino K., Furukawa K. (2017). Non-neural granular cell tumor of the uterine corpus mimicking uterine leiomyoma: A case report. Mol. Clin. Oncol..

[7] AAbrikossoff A. (1926). Über Myome. Virchows Arch..

[8] Elkousy H., Harrelson J., Dodd L., Martinez S., Scully S. (2000). Granular Cell Tumors of the Extremities. Clin. Orthop. Relat. Res..

[9] Lazar A.J.F., Fletcher C.D.M. (2005). Primitive Nonneural Granular Cell Tumors of Skin. Am. J. Surg. Pathol..

[10] Chen S.Y., Sadanand A., A Dillon P., He M., Dehner L.P., Leonard D.S. (2020). Non-Neural (S-100 Negative) Bronchial Granular Cell Tumor Causing Acute Respiratory Failure. Fetal Pediatr. Pathol..

[11] LeBoit P.E., Barr R.J., Burall S., Metcalf J.S., Yen T.S.B., Wick M.R. (1991). Primitive Polypoid Granular-Cell Tumor and Other Cutaneous Granular-Cell Neoplasms of Apparent Nonneural Origin. Am. J. Surg. Pathol..

[12] Rehan S., Paracha H., Masood R., Wang R. (2021). Granular cell tumor of the abdominal wall, a case report and review of literature. AME Case Rep..

[13] McGhan L.J., Wasif N., Young S.W., Collins J.M., McCullough A.E. (2012). Granular-cell tumor of the anterior abdominal wall. Radiol. Case Rep..

[14] Bandyopadhyay D., Sen S., Bandyopadhyay J.P. (2006). Granular cell tumour on vaccinationscar in a young girl. Indian J. Dermatol..

[15] Weiss S.W., Goldblum J.R. (2001). Granular cell tumour. Enzinger and Weiss’s Soft Tissue Tumors.

[16] Mangan S.H., Ng J.Y., Townend P. (2024). A Rare Case Report of Granular Cell Tumour of the Abdominal Wall and a Review of the Literature. Cureus.

[17] Gokaslan S.T., Terzakis J.A., Santagada E.A. (1994). Malignant granular cell tumor. J. Cutan. Pathol..

[18] Klima M., Peters J. (1987). Malignant granular cell tumor. Arch. Pathol. Lab. Med..

[19] Chelly I., Bellil K., Mekni A., Bellil S., Belhadjsalah M., Kchir N., Haouet S., Zitouna M.M. (2005). Malignant granular cell tumor of the abdominal wall. Pathologica.

[20] Ravich A., Stout A.P., Ravich R.A. (1945). Malignant Granular Cell Myoblastoma Involving the Urinary Bladder. Ann. Surg..

[21] Gamboa L.G. (1955). Malignant granular cell myoblastoma. AMA Arch. Pathol..

[22] Lack E.E., Worsham R.G.F., Callihan M.D., Crawford B.E., Klappenbach S., Rowden G., Chun B. (1980). Granular cell tumor: A clinicopathologic study of 110 patients. J. Surg. Oncol..

[23] Cichoń M., Wańczyk-Dręczewska B., Placek W., Biernat W., Owczarczyk-Saczonek A. (2023). Abrikossoff’s tumor in a 23-year-old womanunsuccessfully treated with cryosurgery. Otolaryngol. Polska.

[24] Ardeleanu V., Jecan R.C., Moroianu M., Teodoreanu R.N., Tebeica T., Moroianu L.A., Bujoreanu F.C., Nwabudike L.C., Tatu A.L. (2023). Case report: Abrikossoff’s tumor of the facial skin. Front. Med..

[25] Bitar M., Al Afif K.A., Fatani M.I. (2011). Granular cell tumor: Case report. J. Saudi Soc. Dermatol. Dermatol. Surg..

[26] Pathology Outlines. https://www.pathologyoutlines.com/.

